# Porokeratoses—A Rare Group of Dermatoses

**DOI:** 10.3390/medicina60111876

**Published:** 2024-11-16

**Authors:** Agnieszka Anderska, Agnieszka Kaczmarska-Such, Ewelina Mazur, Adam Reich

**Affiliations:** 1 4th Military Clinical Hospital with Polyclinic SPZOZ, 53114 Wroclaw, Poland; agnieszka@anderski.pl; 2 Doctoral School, University of Rzeszow, 35310 Rzeszow, Poland; agnieszkakacz70@gmail.com (A.K.-S.); mazur.eveline@gmail.com (E.M.); 3 Department of Dermatology, Institute of Medical Sciences, Medical College of Rzeszow University, 35010 Rzeszow, Poland

**Keywords:** porokeratoses, abnormal keratinization, malignant transformation

## Abstract

Porokeratoses represent a rare group of skin diseases characterized by abnormal keratinization. The condition may have a genetic background and can be triggered by environmental factors, including UV exposure and infections. Several clinical variants of porokeratosis can be distinguished, including Mibelli’s porokeratosis, disseminated superficial actinic porokeratosis, superficial disseminated porokeratosis, and porokeratosis palmaris plantaris et disseminata. Diagnosis is established based on clinical and histopathological examination, dermatoscopy, and reflectance confocal microscopy. Various treatment options are available, including topical combination therapy with cholesterol and statins, topical retinoids, cryotherapy, laser therapy, and surgical excision of lesions, but none are fully effective. The success of these treatments can vary significantly based on the specific type of porokeratosis and individual patient characteristics, with many outcomes falling short of expectations. Since the disease is considered a precancerous condition, patients with porokeratosis should remain under regular dermatological control.

## 1. Introduction

Porokeratoses are a rare, heterogeneous group of skin conditions involving keratinization disorders. In the clinical picture, they usually initially take the form of a papule, which evolves into ring-shaped, single, or multiple plaques spreading centrifugally with atrophy in the central part and mild desquamation at the periphery. In addition, porokeratoses are marked by characteristic histopathologic changes called cornoid lamella, which are columns of parakeratotic cells and derive from the abnormal granular layer of the epidermis [1].

The etiology of porokeratoses has yet to be fully understood. It is assumed that this disease has a genetic background and is either caused by de novo mutations or is inherited in an autosomal dominant pattern. Many types of porokeratosis have been distinguished, which differ in their clinical presentation. Commonly recognized variants include disseminated superficial actinic porokeratosis (DSAP), porokeratosis of Mibelli (PM), disseminated superficial porokeratosis (DSP), linear porokeratosis (LP), and porokeratosis palmaris plantaris et disseminata (PPPD). Other types of porokeratoses may also occur, but their incidence is very low [2].

Porokeratosis is considered a precancerous condition, as all its variants have the potential for malignant transformation [3]. The incidence of developing skin cancers within the lesions depends on the type of porokeratosis, with the lifetime incidence ranging from 6.4% to 19%, with squamous cell carcinomas being the most common [4,5]. For this reason, patients with porokeratosis should remain under rigorous dermatological control.

## 2. Epidemiology

The exact incidence is unknown [6]. Morbidity is mainly reported among adults in the 4th and 5th decades of life, but some cases have also been described in children [3]. Women and men are affected with similar frequency, but depending on the type, one of the sexes may predominate [7]. PM and PPPD seem to be more common among men, while DSAP and LP are more common among women [8].

## 3. Etiology and Pathophysiology

**Figure 1:**
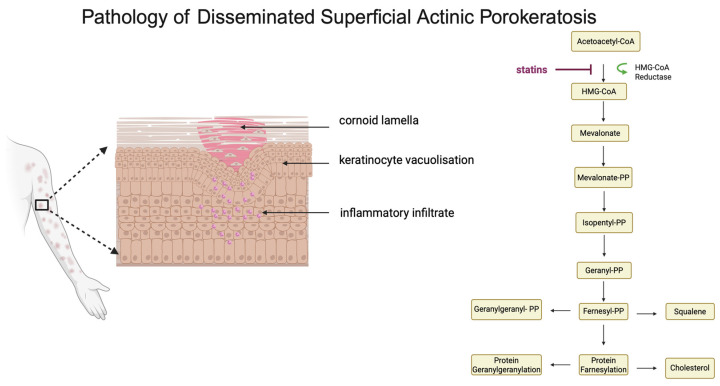
Histopathological features of disseminated superficial actinic porokeratosis (DSAP) include the presence of a cornoid lamella, epidermal atrophy, and lymphocytic infiltration in the dermis. The mevalonate pathway, responsible for cholesterol biosynthesis, is linked to the pathogenesis of porokeratosis.

Although the first reports on this group of dermatoses are dated back to 1899, their etiology is still not fully understood [9]. Some of the reported cases are familial, indicating a genetic basis for the disease, which is inherited in an autosomal dominant manner [10]. The current studies were able to establish that porokeratosis may be caused by mutations occurring in the mevalonate pathway, more specifically, in the genes for mevalonate decarboxylase, mevalonate kinase, phosphomevalonate kinase, and farnesyl diphosphate synthase (Figure 1), which are involved in the biosynthesis of cholesterol and sterols [11]. Changes in this pathway have been observed among 16% of patients with the acquired form of DSAP and among 33% patients with the familial form of DSAP [12]. Porokeratoses may also result from de novo mutations [13]. UV exposure is an example that triggers abnormalities in epidermal keratinization [14]. It promotes loricrin synthesis and abnormal keratinocyte death, which affect keratinocyte terminal differentiation and ultimately result in dyskeratosis [15]. Furthermore, the illness is linked to immunodeficiency in almost half of patients, including cancer patients and transplant recipients [16]. Drugs, trauma, or infections could be additional etiological factors. Among infections, most associated with porokeratosis appear to be human papillomavirus (HPV) [17], herpes virus (HSV) [18], hepatitis C virus (HCV) [19], and human immunodeficiency virus (HIV) [20]. On the other hand, furosemide, hydrochlorothiazide, and biologic drugs (etanercept, certolizumab) have been identified as causative factors of porokeratosis [21,22].

The primary tests for diagnosing porokeratoses are clinical and histological examinations, as well as dermatoscopy [23]. The diagnostic feature of all porokeratoses is the presence of a coronoid plaque, which is the clinically visible raised edge of the lesion. On histopathology view, the cornoid lamella is a parakeratotic column that covers a small vertical zone of dyskeratotic and vacuolated cells within the epidermis [24]. Moreover, prevalent characteristics of dermatoscopy is a hyperkeratotic edge of various colors—white, yellow, or brown—which often demonstrates a characteristic doubled shape. Various changes depending on the stage of the disease can be observed in the central part of the lesion. Dot- and papule-type vessels are found in active lesions, with blue-gray dots and papules present in healing lesions and those of a white color, mainly polarity-dependent lines, in descending lesions. Furthermore, it may be beneficial to use dermatoscopy enhanced with ink or alternative dyes. After cleansing the lesion with alcohol, the dye adheres to the surface, highlighting the distinct dermatoscopic structures and facilitating the differentiation of porokeratosis from other dermatological conditions [25].

Histopathological examination reveals narrow, closely packed columns of parakeratotic cells, known as keratinized plaques. The granular layer beneath the keratinized plaque is often atrophic or thinning, and cells in the lower layers of the epidermis may show vacuolization changes. In addition, nonspecific lymphocytic infiltrates may be present in the dermis [26]. It is also essential to be mindful of the differential diagnosis of porokeratosis, which involves skin diseases such as lichen planus, psoriasis, solar keratosis, and granuloma annulare [27].

## 4. Clinical Variants of Porokeratosis

### 4.1. Disseminated Superficial Actinic Porokeratosis (DSAP)

**Figure 2:**
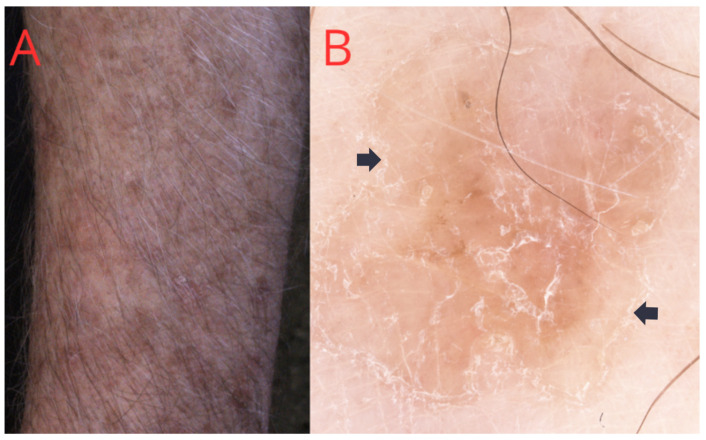
(**A**) Disseminated superficial actinic porokeratosis (DSAP): diffuse, small plaques on the man’s forearm. (**B**) Dermatoscopic view—a peripheral white line with a doubled edge, characteristic of porokeratosis (black arrow).

DSAP is the most common form of porokeratosis, representing about 56% of all porokeratosis, and is associated with UV radiation [28]. The skin lesions primarily appear on exposed body regions, including the backs of the hands, lower thighs, and vertical portions of the forearms (Figure 2), but they can additionally appear on the back and, in approximately 15% of patients, on the face. DSAP is more common in women, with the highest incidence reported in Australia (probably due to high sun exposure) and among those with a history of phototherapy [7]. In the differential diagnosis of DSAP with other types of porokeratosis, sun exposure is crucial. Moreover, DSAP is often associated with solar keratosis. The lesions are demarcated, diffuse, and annular, measuring 0.5–1.5 cm in size, featuring a prominent raised, gently exfoliating hyperkeratotic border, along with a slightly atrophic central area [29].

### 4.2. Porokeratosis of Mibelli (PM)

**Figure 3:**
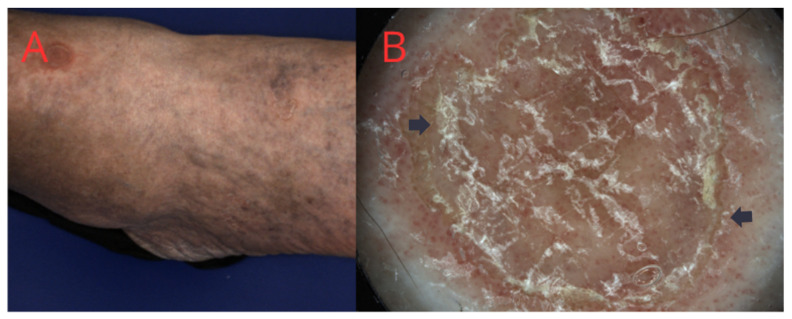
(**A**) Porokeratosis of Mibelli—two plaques on the man’s arm and forearm. (**B**)Dermatoscopic view: characteristic peripheral white-yellow line with a doubled edge (black arrow) and multiple dot-type vessels.

PM is the second most common form of porokeratosis, typically acquired and more prevalent in men. In contrast to DSAP, which is more prevalent in older people, Mibelli’s porokeratosis frequently affects children and young adults [30]. Immunosuppression is the likely causative factor; in addition, it is common in patients suffering from hepatitis B, bone marrow transplant recipients, or chronic users of topical corticosteroids [31,32]. The lesions generally progress slowly and are asymmetric, primarily affecting the anterior surface of the lower extremities, though they can also appear on the face, trunk, genitals, or even nail palate (Figure 3). Initially, a small, slightly itchy papule enlarges over time, reaching up to several centimeters in size. It may also present as multiple papules or macules resembling DSAP, featuring central atrophy and raised keratinized edges. Several variants of this porokeratosis include linear, giant, or hyperkeratotic forms [33].

### 4.3. Disseminated Superficial Porokeratosis (DSP)

**Figure 4:**
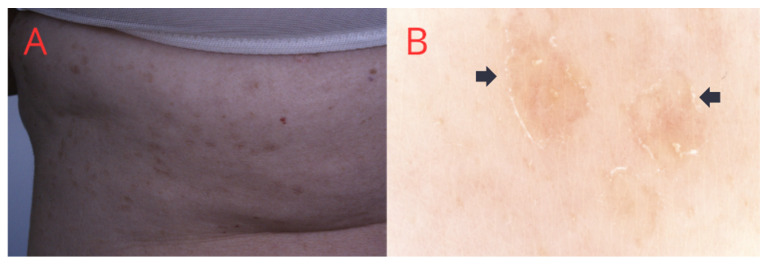
(**A**) Disseminated superficial porokeratosis—multiple small erythematous plaques on the woman’s back. (**B**) Dermatoscopic view—a peripheral, interspersed white line with a doubled edge and centrally located linear vessels and single keratotic plug at the openings of hair follicles (black arrow).

DSP is a rare form of porokeratosis, and the average age of onset is between the fourth and fifth decades of life. Women are more commonly affected. Immunosuppression is considered the most important triggering factor [34]. The clinical picture resembles DSAP (Figure 4), but the lesions appear not only on UV-exposed skin [35]. DSP occurs mainly on the trunk, upper and lower extremities, genitalia, or, rarely, the oral cavity [36].

### 4.4. Porokeratosis Palmaris, Plantaris et Disseminata (PPPD)

**Figure 5:**
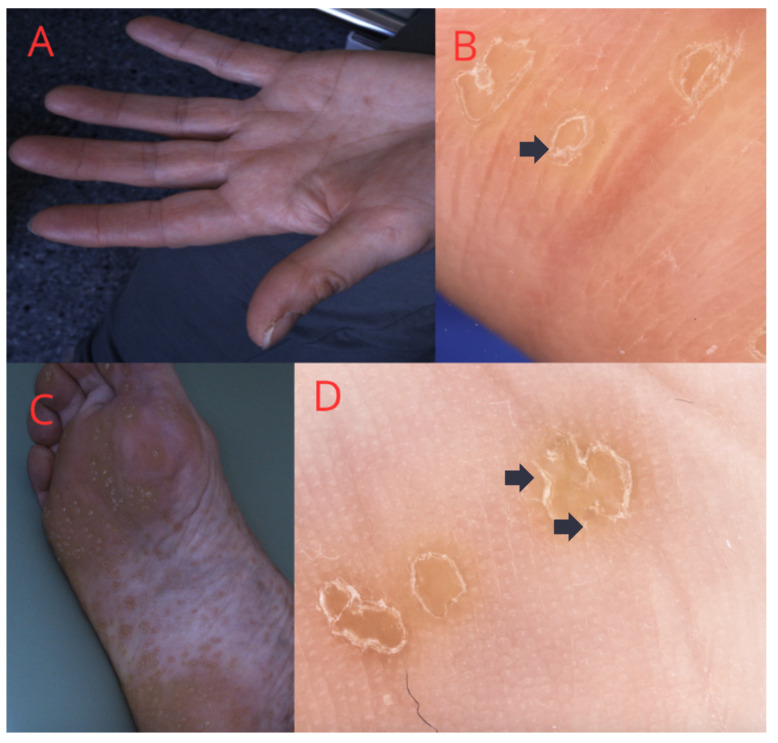
(**A**–**C**) Porokeratosis palmaris, plantaris et disseminata: multiple small plaques on the woman’s hands and feet. (**B**–**D**) Dermatoscopic view—peripheral white lines with a doubled edge (black arrow) with a central structureless area, characteristic of porokeratosis.

PPPD is a rare subtype that primarily affects adolescents and young adults, especially young men. Initially, PPPD is limited to the hands and feet and then spreads to other parts of the body. It can also affect the nails and mucous membranes (Figure 5). Lesions may take the form of keratotic papules or be characterized by a picture similar to DSAP. However, unlike DSAP, PPPD lesions can also occupy non-UV-exposed skin, but interestingly, exacerbation of the disease is often observed during the summer months [37].

### 4.5. Linear Porokeratosis (LP)

LP is a form of porokeratosis similar to DSAP, distinguished by the arrangement of lesions in a linear way, usually along Blaschko’s lines. Most cases manifest in childhood and localize on the extremities [38]. It is characterized by a high risk of malignant transformation reaching up to 20%, most often to squamous cell carcinoma [10].

Other types of porokeratosis such as porokeratosis ptychotropica or follicular porokeratosis also occur, but their incidence has been described as very low [39].

## 5. Malignant Transformation

Porokeratoses are known to be a precancerous condition that can potentially lead to skin cancer, primarily squamous cell carcinoma, but also less frequently basal cell carcinoma and occasionally melanoma [5]. Porokeratoses can also predispose to the development of other conditions, such as Bowen’s disease and skin horns [40]. A higher risk of developing skin cancers is noted with longer disease duration, more severe course, large lesion size, and in immunocompromised and elderly patients [41]. Depending on the type of porokeratosis, the risk of developing cancer varies. The highest is noted in giant and linear porokeratosis [8,10].

In a study by Novice et al. [4], 110 patients with porokeratosis were examined, and the incidence of malignancy in this group was 6.4%. In contrast, in a study by Sasson et al. [42], malignant transformation was observed in 7.6% of PM cases, in 3.4% of DSAP cases, in 19% of linear porokeratosis cases, and in 9.5% of PPPD cases. However, due to the rarity of porokeratoses, studies on large patient populations are lacking to accurately estimate the rate of malignant transformation in different types of porokeratosis [1].

## 6. Diagnostics

The medical history and clinical picture in many cases are sufficient to make the diagnosis [23]. On dermatoscopic examination, a characteristic keratotic plaque in the form of a peripheral white line with a doubled edge is present in more than 90% of patients with porokeratosis. It may be accompanied by gray-brown/blue dots and papules, discoloration, or white lines. The presence of central brown pigmentation, dot-shaped or glomerular vessels, scales, and small petechiae is also characteristic [1]. Biopsy and histopathological examination are reserved primarily for atypical lesions and questionable cases. It is important to take material from the elevated border of the lesion so that the corneal lamina can be visualized [7]. The differential diagnosis of porokeratoses should include psoriasis, cutaneous tuberculosis, Bowen’s disease, candidiasis, allergic dermatitis, and solar keratosis [25]. Special attention should be paid to lichenoid lesions–lichen planus and benign lichenoid keratosis [43,44,45].

## 7. Treatment

Topical statins, such as simvastatin and lovastatin, show potential therapeutic activity in the treatment of porokeratoses (Table 1). They prevent the excessive accumulation of mevalonate pathway metabolites by inhibiting HMG-CoA reductase. DSAP patients have demonstrated significant skin improvements after using ointments with 2% simvastatin or lovastatin and 2% cholesterol, but clinical improvement was observed after six weeks of treatment [46,47,48].

Another topical substance is 5-fluorouracil, which blocks DNA synthesis and has a cytotoxic effect on rapidly dividing cells. By causing the inhibition of excessive cell proliferation, it finds its use in the treatment of porokeratosis [49]. In some patients, 5-fluorouracil causes inflammatory changes in and irritation of the skin, but this is usually transient, and treatment has good results [50]. It also shows high efficacy in combination with other drugs, such as imiquimod or calcipotriol [51]. The mechanism of action of imiquimod is the production of cytokines and, therefore, the stimulation of the resistance response, which is often impaired in porokeratosis [52]. Its greatest efficacy has been reported in the porokeratosis of Mibelli, as well as a form involving the palms and soles [53].

Vitamin D derivatives show their therapeutic effect by modulating the differentiation and proliferation of keratinocytes [54]. The treatment is generally effective with minimal side effects, but there is potential for even better results in some cases. Combining them with retinoids significantly improves the treatment effect [55,56]. Retinoids in monotherapy also provide good results, both in topical and systemic treatment. It is worth mentioning that topical treatment carries a lower risk of side effects [57].

Diclofenac, as a selective COX-2 inhibitor, has also found use in the treatment of porokeratoses, although the mechanism is not fully understood [58]. Most sources report good efficacy with this method, but full remission is rarely achieved, and redness and pruritus are among the more common complications [59]. Other topical medications that are effective in the treatment are 0.7% cantharidin and 0.015% ingenol mebutate (currently not available), which work particularly well in cases of excessive keratosis [60].

There are also isolated reports of the efficacy of topical steroids in patients with porokeratosis and co-occurrent pruritus [61]. On the other hand, the use of systemic steroids is associated with the development of porokeratoses, most likely due to the immunosuppressive effects of systemic glucocorticosteroids [62].

Cryotherapy is a method of freezing individual lesions with liquid nitrogen. Its good efficacy, especially against DSAP, is reported in the literature [63]. Laser therapy is a good alternative when other methods are ineffective. The CO_2_ laser shows good efficacy but leaves scarring in some cases. A better option seems to be the Q-switched ruby laser, which does not leave scars and has a good healing effect. However, it requires longer follow-up [64,65,66].

Photodynamic therapy is effective in patients with a small number of skin lesions. The photosensitizers used are MAL (methyl aminolevulinate) or ALA (5-aminolevulinic acid), which are captured by atypical keratinocytes after topical application. Following a series of photochemical reactions, the cells are destroyed by exposure to light [67]. The surgical excision of lesions has little use in this indication and is mainly limited to small lesions. It also causes discoloration or scarring. Skin grafting is primarily used, but this method is rarely described in the literature [68,69].

**Table 1:** Summary of treatment results of different types of porokeratosis according to selected studies.

Authors	Study Type	Type of Porokeratosis	Number of Patients	Treatment	Outcome	References
Ugwu N, Choate KA, Atzmony L	Case report	DSAP	1	2% Cholesterol + 2% Lovastatin ointment	Significant improvement in scaling and erythema after 4 weeks of therapy.	[46]
				2% Cholesterol ointment	No improvement after 4 weeks of therapy.	
				2% Lovastatin ointment	Remarkable decrease in scaling after 4 weeks. Complete response after 6 weeks of therapy.	
Atzmony L, Lim YH, Hamilton C, Leventhal JS, Wagner A, Paller AS, Choate, KA.	Original article	DSAP	5	2% Cholesterol + 2% Lovastatin ointment	After 4 weeks of therapy, there was a marked decrease in erythema, scaling, and size of visible lesions. After 3 months, only small erythematous macules.	[48]
		PPPD	2		A remarkable decrease in scaling after 3–4 weeks. After 5 weeks, decrease in thickness and erythema.	
		LP	2		A decrease in scaling after 4 weeks and moderate decrease in erythema in 6–8 weeks.	
Buhle AC, Fagan KK, Johnson NM, Grider DJ	Case report	LP	1	2% Cholesterol + 2% Lovastatin cream	Partial resolution of the lesions.	[70]
McDonald SG, Peterka ES	Case report	PM	1	5-Fluorouracil, cream and occlusion with petrolatum	After 5 weeks, partial improvement in the lesions; treatment for another 2 weeks resulted in significant improvement. After two years of follow-up, no recurrence was found.	[71]
Gongale SP, Hajare SA, Mukhi JI, Singh RP	Case report	labial PM	1	5-Fluorouracil, cream	After 16 weeks almost, complete resolution of the lesions.	[72]
Porter WM, Menagé H. Du P, Philip G, Bunker CB	Case report	PM	1	5-Fluorouracil, cream	Good response to treatment after 4 weeks.	[73]
Venkatarajan S, LeLeux TM, Yang D, Rosen T, Orengo I	Case report	PM	1	5-Fluorouracil + 5% Imiquimod, cream	After 16 weeks, the skin lesions disappeared, leaving mild residual hyperpigmentation.	[50]
Yeh JE, Nazarian RM, Lorenzo ME	Case report	PM	1	5% 5-Fluorouracil + 0.005% Calcipotriene, cream	Complete resolution of the lesions.	[74]
S Jain	Case report	PM	1	5% Imiquimod, cream	After 6 weeks, complete resolution of the lesions. After a year of follow-up, no recurrence was found.	[75]
Agarwal S, Berth-Jones J	Case report	PM	1	5% Imiquimod, cream	No improvement after 3 months.	[52]
				5% Imiquimod, cream under occlusion with an adhesive polythene dressing	After 5 weeks, complete resolution of the lesions. After a year of follow-up, no recurrence was found.	
Böhm M, Luger TA, Bonsmann G	Case report	DSAP	1	0.0004% Tacalcitol, ointment	At the beginning of treatment, the pruritus disappeared, and after 5 months, the lesions faded completely, with no recurrence.	[76]
Nakamura Y, Yamaguchi M, Nakamura A, Muto M	Case report	DSAP	1	0.005% Calcipotriol ointment	After 3 months slight improvement.	[56]
				Calcipotriol + 0.1% Adapalene, gel	After 3 months, the skin lesions improved substantially, leaving only slight hyperpigmentation.	
Roziewska D, Szczerkowska-Dobosz A, Komorowska O.	Case report	DSP	1	Isotretinoin, gel, then Tretinoin, liquid and cream	After 18 months, a marked improvement in the lesion; the hyperkeratotic border disappeared and the lesion faded	[77]
				Tretinoin, cream, then Adapalene, cream	Continuation of treatment for about 1.5 years; complete regression of lesions.	
Hong JB, Hsiao CH, Chu CY.	Case report	LP	1	Acitretin 30 mg/d, oral	After one month, reduction in erythema and hyperkeratosis; after 7 months, no more cornoid lamella in biopsy	[53]
Marks S, Varma R, Cantrell W, Chen, SC Gold, M Muellenhoff M.,Elewski B.	Multicenter Study	DSAP	17	3% Diclofenac gel	Partial improvement in lesions without complete remission.	[78]
Vlachou C, Kanelleas AI, Martin-Clavijo A, Berth-Jones J.	Case series	DSAP	8	3% Diclofenac gel	Among 5 patients, no improvement; in 3 patients, partial response and reduction in pruritus.	[59]
Kluger N, Dereure O, Guilhou JJ, Guillot B.	Case report	PP	1	3% Diclofenac gel + 0.1% Diflucortolone ointment	Partial improvement in the lesion and significant reduction in pruritus.	[58]
Levitt JO, Keeley BR, Phelps, RG.	Case report	PM	2	0.7% Cantharidin	Within 1 week, each lesion cleared completely; however, in both cases, post-inflammatory erythema remained, even after 6 months.	[55]
Ghahartars M, Zahraei SAH, Sari Aslani F, HadibarhaghtalaBM, Parvizi MM.	Case report		1	Cryotherapy	The lesions disappeared and complete cure occurred after 4 weeks. No recurrence in 6 months.	[79]
Fustà-Novell X, Podlipnik S, Combalia A, Morgado-Carrasco D, Ferrando J, Mascaró J, Jr JM, Aguilera P	Review	PP	2	Photodynamic therapy, MAL-PTD	Pruritus relief, decrease in desquamation, and a halt in plaque growth. Complete clearance of the plaques could not be achieved.	[80]
Nayeemuddin FA, Wong M, Yell J	Case series	DSAP	3	Photodynamic therapy, ALA-PTD	No significant improvement	[81]
Curkova AK, Hegyi J, Kozub P, Szep Z, D’Erme AM, Simaljakova M	Case report	LP	1	Photodynamic therapy, MAL-PDT	After 3 cycles, significant improvement; the lesions were pink-colored with subtle scaling.	[67]
Lecamwasam K, Skellett AM, Levell NJ	Case report		1	Photodynamic therapy, MAL-PDT	After 4 cycles, 90% of the lesions had flattened and faded after 3 months.	[82]
Mu X, Li W, Zhang M, Yang, C, Yang X, Li D, Ding Y.	Case report	EDP	1	Tofacitinib 5 mg	Significant improvement after 1 month, leaving only brown pigmentation on the erythema of the extremities.	[83]

DSAP—disseminated superficial actinic porokeratosis, LP—linear porokeratosis, ALA-PTD—aminolevulinic acid photodynamic therapy, MAL-PTD—methyl aminolevulinate photodynamic therapy, EDP—eruptive disseminated porokeratosis, PM—porokeratosis of Mibelli, PP—porokeratosis ptychotropica.

## 8. Conclusions

Porokeratoses are a group of skin diseases that, depending on the type, differ in their clinical picture. Due to the risk of malignant transformation, it is extremely important to recognize it early and start the appropriate treatment. Of all the treatment methods, the most promising seems to be a combination of topical 2% lovastatin and 2% cholesterol, photodynamic therapy, and QSRL laser therapy. If the therapeutic effect is unsatisfactory, the various methods can be combined and tailored to the individual needs of each patient.

## Abbreviations

ALA-PTD	aminolevulinic acid photodynamic therapy
DSAP	disseminated superficial actinic porokeratosis
EDP	eruptive disseminated porokeratosis
LP	linear porokeratosis
MAL	methyl aminolevulinate
MAL-PTD	methyl aminolevulinate photodynamic therapy
PM	porokeratosis of Mibelli
PP	porokeratosis ptychotropica
PPPD	porokeratosis palmaris, plantaris et disseminata
QRSL-Q	switched ruby laser
UV	ultraviolet light
